# Analysis of Speed Performance In Soccer by a Playing Position and a Sports Level Using a Laser System

**DOI:** 10.2478/hukin-2014-0120

**Published:** 2014-12-30

**Authors:** Amelia Ferro, Jorge Villacieros, Pablo Floría, Jose L. Graupera

**Affiliations:** 1 Faculty of Physical Activity and Sport Sciences-INEF. Technical University of Madrid (UPM), Spain.; 2 Faculty of Sport. Pablo de Olavide University (UPO), Seville, Spain.; 3 Department of Biomedical Sciences II. University of Alcalá (UAH), Madrid, Spain.

**Keywords:** soccer, sprint, velocity, 30 m test, kinematics

## Abstract

The purpose of this study was to determine the kinematic variables that identify the quality of velocity in soccer players at different competitive levels and playing positions. This study had two independent variables: 1) a competitive level (competitive and non-competitive players); and 2) a playing position, with four levels (central defenders, wide defenders/midfielders, central midfielders and forwards). Forty-two soccer players took part in a 30 m sprint-test, which was measured using a laser sensor-type 1 (LDM301-Jenoptik) at 2000 Hz. Absolute and relative times, average velocities and absolute and relative maximum velocities over 10 m sections were analyzed at 200 Hz with BioLaserSport^®^. There were no significant differences in average velocity between competitive and non-competitive players; however, the former reached a greater maximum velocity in the 10–20 m section. Average velocity in the 0–10 m section identified specificity among playing positions in competitive players. The forwards were the fastest followed by the central midfielders, the wide defenders/midfielders and the central defenders. No differences were found among the non-competitive players. Average velocity over the 0–10 meter section may be an important indicator when assigning a playing position for competitive players. These results support the use of more accurate systems, such as a laser system, to identify soccer players’ speed qualities (including maximum velocity) during short sprints.

## Introduction

Short sprints are very common in many team sports including soccer, rugby, basketball, hockey, etc. High intensity activities are defined as those that are carried out from 5.3 to 6.3 m·s^−1^ (Di Salvo et al., 2007) and, specifically, sprinting activities are those performed at over 7 m·s^−1^ (Rampinini et al., 2007). Scientific data substantiating the importance of power and velocity in soccer are scarce (Faude et al., 2012). Studies on sprinting indicate that during a match a player is standing for 19.5% of the total time, walking for 41%, running at a low intensity for 30% and at a high intensity (> 5 m·s^−1^) for 8.7%, of which sprinting (> 8.3 m·s^−1^) accounts for 1.4% (Mohr et al., 2003). Krustrup et al. (2005) observed that in the best Danish Female League, 4.8% of the total time was spent in high intensity running (> 5 m·s^−1^). The importance of sprinting is that it is utilized at key moments during a match, allowing a player to escape from his opponent and/or to reach a free zone to shoot on the goal or to make a decisive pass (Faude et al., 2012). Short-sprinting performance might be an important determinant of match-winning actions (Cometti et al., 2001). The importance of analyzing the intensity and frequency of movements during a match lies in the fact that approximately 98% of the distance covered by players occurs when they are not in possession of the ball (Reilly and Thomas, 1976). Di Salvo et al. (2007) observed that only 1.2 to 2.4% of the running distance in match play is covered with possession of the ball.

During high intensity sprints (> 6.3 m·s^−1^) the players covered between 9.9 – 32.5 m of the total distance (average of 19.3 ± 3.2 m) over the course of 17.3 ± 7.7 (range of 3 – 40) sprints (Di Salvo et al., 2007). During the European Champions League and UEFA Cup, the average number of sprints (> 7 m·s^−1^) varied by a playing position: wide midfielders (35.8 ± 13.4), forwards (attackers) (30.0 ± 12.0), wide defenders (29.5 ± 11.7), central midfielders (23.5 ± 12.2) and central defenders (17.3 ± 8.7) (Di Salvo et al., 2010). In terms of the section analyzed (0–5 m, 5.1–10 m, 10.1–15 m, 15.1–20 and > 20 m), players were found to carry out more sprints in the first section, with differences according to a playing position in the English Premier Soccer League (Di Salvo et al., 2009).

Faude et al. (2012) analyzed the influence of speed and power abilities on goal situations among professional soccer players participating in the First German National League. They observed that 83% of the goals were preceded by one, two or more powerful actions of the scoring players (62% of 360 goals) or of the assisting players (55% of 322 goals). Straight sprinting was the most frequent and dominant powerful action in decisive offensive situations followed by jumps, cuts and changes of direction. Most actions by the scoring player were straight sprints (45% of all analyzed goals). Straight sprints by the scoring players (61%) were conducted without an opponent (41.6%) and without the ball (75%), both in reference to their total straight sprints. Similarly, battling against an opponent and/or with the ball may slow down velocity. The most frequent action of the assisting players was the straight sprint (67%), mostly conducted without an opponent (40.5%), but with the ball (64%). Considering the relevance of straight sprinting in decisive situations, the authors recommended that fitness training and testing should include such sprints.

Other studies focused on analyzing the differences in performance between elite and sub-elite soccer players and observed that the elite players reached faster times over 15 m, 25 m and 30 m sprint distances than did the sub-elite players (Reilly et al., 2000). Cometti et al. (2001) observed that professional first division and second division players ran faster over 10 m than amateur players in a 30 m test. With regard to speed, some authors point to several aspects that should be assessed, such as acceleration and maximum velocity over an average of three repetitions, and they suggested the use of 10 m and 20 m tests, respectively (Sayers et al., 2008). Other authors used a 30 m test (Gil et al., 2007; Taskin, 2008), a 30 m test every 10 m (Wisloff et al., 2004) or different repeated sprint tests (Kaplan, 2010; Kaplan et al., 2009; Rampinini et al., 2009; Aziz et al., 2008).

In the last few years researchers have developed new technology to obtain accurate “real time” results for the variables related to the positions and velocity of soccer players on the field. As well as using multiple timing systems such as photoelectric cells, Global Positioning Satellite devices (GPS) (Barbero-Álvarez et al., 2010) and the new Prozone system (Di Salvo et al., 2006), laser technology has been adapted to measure running speed accurately and immediately (Dickwach et al., 1994; Harrison et al., 2005). The laser system can record positions and velocities at high frequencies, ranging from 100 – 200 Hz for these tests, achieving optimal accuracy, and has been used by some authors (Adamczewski and Perlt, 1997; Brüggemann et al., 1999; Delecluse et al., 2005; Zwierko and Lesiakowski, 2007).

Based on the aforementioned articles, the present study considered specific sprint characteristics in a game, according to the field position and competitive level. The laser system used in this analysis is an innovation that could make it possible to identify differences in sprint velocities not detected with other customary techniques and may be able to guide coaches’ decision making. Therefore, the purpose of this study was to analyze whether using the 30 m sprint test, registered with an accurate laser system, could identify the different playing positions in soccer players at different competitive levels.

## Material and Methods

### Approximation method

The present study compared performance of soccer players from different playing positions and competitive levels in a 30 m sprint test to observe whether there was a difference in the quality of velocity among the different positions. Performance was assessed with a 30 m sprint test using a laser system capable of recording the soccer player’s position and velocity at a sampling frequency of 2000 Hz. Forty-two soccer players participated in the study and were divided into eight groups according to their competitive level (competitors and non-competitors) and playing position (central defenders, wide players, including wide defenders and wide midfielders, central midfielders and forwards). Thirty dependent variables were defined and measured in the 0–10, 10–20, 20–30, 0–20, 0–30 and 10–30 m sections obtaining absolute and relative times, average and maximum absolute and relative velocities. We expected to find differences among the groups that would make it possible to define the variables that determine speed performance in soccer players. These data could help coaches to determine which velocity characteristics are most suitable for each playing position.

### Participants

A total of 42 university students from the Physical Activity and Sport Sciences program participated in this study. They were divided into two groups based on their performance level: competitive and non-competitive soccer players. The competitive group consisted of 21 soccer players who competed in the national level junior, first or second regional divisions. The non-competitive group consisted of 21 physically active subjects who played soccer 2–3 days a week. Each group was further divided into four subgroups according to their position on the field: 11 defenders, 13 wide defenders and wide midfielders, 11 central midfielders and 7 forwards (Table 1). These subgroups were based on Di Salvo et al. (2010), but we regrouped wide defenders and wide midfielders because both performed offense and defense actions, and their movements were on the flank of the field. None of the participants was suffering from injuries at the time of the test. The study was approved by the Ethics Committee of the Technical University of Madrid. The volunteer participants were informed about the study’s purpose and the tests that they were going to perform and signed their informed consent prior to the commencement of the study. All of them wore comfortable sports gear and soccer boots for the tests.

### Procedures

A laser sensor-type 1 (*LDM301, Jenoptik, Germany)* integrated into a *Kinematic analysis system in real-time for the training and the sports competitions* (Ferro and Floría, 2010) *BioLaserSport*^®^ (Ferro, 2012) was used. The laser sensor has a measuring range of 0.5 to 300 m on natural surfaces, an accuracy of ± 0.06 m for measurements of 2 kHz and a resolution of 0.001 m. The test distances were measured with a *Stanley TLM160i (Mechelen, Belgium)* laser calibrated according to the ISO standard with a range of 0.05 to 60 m, accuracy of ± 0.0015 m and a visualized minimal unit of 0.001 m. The data were recorded and processed with a calculus routine designed by the authors using the *DASYLAB v. 10.0* software program (*Data Acquisition System Laboratory* of *National Instruments, Mönchengladbach, Germany*).

The tests were performed on a natural grass pitch on two consecutive days between 10 a.m. and 2 p.m. The meteorological conditions were similar with dry sunny weather, and there were similar conditions on the grass pitch on both days. The test started with a standard 15 min warm-up, which included continuous running, joint mobility, static and dynamic stretching of the lower and upper limbs and 3–4 series of 30 m runs at increasing velocity until a submaximal velocity was reached. The participant then stood at the starting line with the front foot on the line and the trunk behind it. Two posts were placed at each end of the line to make sure that the player’s chest did not cross the line before the start of the test. The starting signal was verbal. Each player performed three 30 m sprints with a 5 min rest period between runs. The laser beam had to hit the player’s back at a height of 1 m from the ground with the horizontality of the beam being controlled. Position data were recorded at a sampling frequency of 2000 Hz. Average data were calculated for each 10 records, obtaining plots and data for positions and velocities to 200 Hz in real time. The data were filtered at a frequency of 3 Hz with a *second-order Butterworth low-pass filter*. The validity and reliability of *BioLaserSport^®^* were analyzed (Ferro et al., 2012) and its reliability was assessed for all parameters presented in this study. The intraclass correlation coefficient (ICC) was ≥ 0.97 for all Vm and ≥ 0.93 for all Vmax. The following variables were calculated:

- *T*: Interval time taken to cover each section (T0–10, T10–20, T20–30, T0–20, T0–30, T10–30) expressed in s.
- *RT*: Interval time taken to cover each section relative to the time over the 30 m (RT0–10, RT10–20, RT20–30, RT0–20, RT10–30). RT (section) = (Tsection × 100)/ T0–30, expressed in percent.
- *Vm*: Average value for the velocity over each section (Vm0–10, Vm10–20, Vm20–30, Vm0–20, Vm0–30, Vm10–30), expressed in m·s^−1^.
- *Vmax*: maximum velocity reached in each section (Vmax0–10; Vmax10–20; Vmax20–30), expressed in m·s^−1^.
- *RV*: Percentage of velocity relative to maximum velocity reached over the 30 m (RV0–10, RV10–20). RV section = (Vmax section × 100)/ Vmax0–30, expressed in m·s^−1^.

### Analysis

A two-way ANOVA was applied using the level of competition (2 levels) and the playing position (4 levels) as factors along with 30 dependent variables. The normality of the variables was tested with the *Kolmogorov-Smirnov Test* and the coefficients of asymmetry and kurtosis were found. Subsequently the *Levene’s test* was applied to analyze the equality of the variances in the competitive level groups and by a playing position and to see if an ANOVA should be performed. Using a one-way ANOVA for the independent variables of the competitive level and playing position, the degree of significance was determined at *p* < 0.05 and η*^2^*, establishing from 0.2 a moderately consistent effect. Lastly, the effect of the interaction among the competitive level and playing positions was studied among the eight groups formed. The level of significance was set at *p* < 0.05. All the calculations were performed with the *SPSS 18.0* program.

## Results

RT0–10 accounted for 42%, RT10–20 for 29% and RT20–30 for 22% of the total time. RV reached 84% in the first 10 m and 95% in 20 m. Tables 2 and 3 show the descriptive data for average velocities, maximum velocities and velocities relative to the maximum, respectively, calculated for the of 0–10, 10–20, 20–30, 0–20, 0–30 and 10–30 m sections.

**Figure 1:**
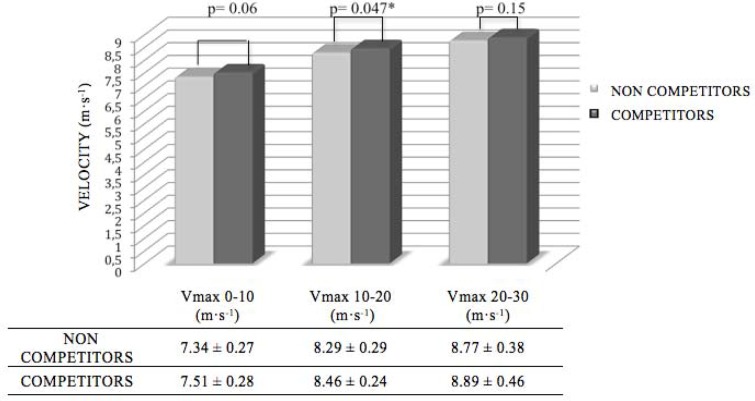
Maximum velocity by the sections among competitors and non competitors (*F = 4.26)

The results of the variables analyzed with the ANOVA in relation to the competitive level showed significant differences for Vmax10–20 (*p* < 0.05) (Figure 1).

**Figure 2:**
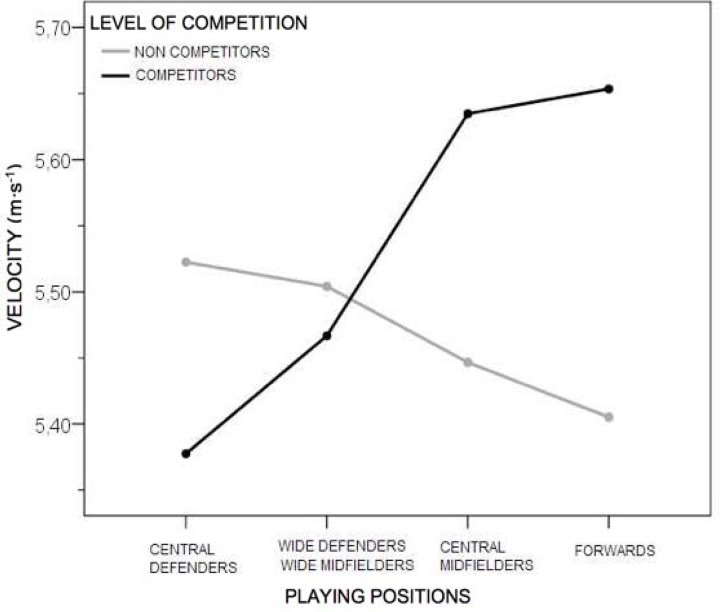
Effect of the interaction among the level of competition and playing positions for Vm 0–10 m

Figure 2 shows the effect of the interaction among the competitive level and playing positions (*p* < 0.05; η^2^ = 0.21). Specificity was evident in the playing position in relation to average velocity in the 0–10 m section with the forwards being the fastest, followed by the central midfielders, wide defenders-midfielders and central defenders, in this order.

## Discussion

The 30 m test has often been used by authors to assess velocity of soccer players (Reilly et al., 2000; Cometti et al., 2001; Wisloff et al., 2004). The results of this study indicated that there were no significant differences in the time T0–30 m between the groups of competitors and non-competitors, which were T0–30: 4.29 ± 0.16 s and 4.34 ± 0.13 s, respectively (*p* = N.S.). The results obtained from elite players by Reilly et al. (2000) were similar to those found in the present study (4.31 ± 0.14 s).

The data on times per section in the competitive players were T0–10: 1.82 ± 0.07 s, T0–20: 3.8 ± 0.1 s and T0–30: 4.28 ± 0.2 s and were consistent with those found by Wisloff et al. (2004), who collected data from a 30 m test with elite players from the first division in Norway, recording similar times in the first section (1.82 ± 0.3 s) but slower times in the second and third sections (3.0 ± 0.3 s and 4.0 ± 0.2 s, respectively). This result seems logical due to the difference in the competitive level between the two groups of soccer players. The time analysis considered in the present study made it possible to discriminate the partial times for the 30 m sprint. In a study carried out with professional first division, second division and amateur French soccer players, Cometti et al. (2001) found that professional players were faster in the first 10 m than amateurs; however, they did not find significant differences between the three groups over the whole distance of 30 m. Analysis of partial times may be necessary to achieve meaningful results, as the soccer players obtain very similar results in a 30 m sprint test, but different times for the first 10 m of the test.

The most innovative aspect of the present study was the possibility of describing the velocity curves over the 30 m sprint with a laser system at a frequency of 200 Hz. Barbero-Álvarez et al. (2010) showed that the GPS system permitted a valid and reliable estimation of maximum velocity when assessing distances between 15 and 30 m. However, it is not the most suitable system to assess velocity performance over shorter distances (Barbero-Álvarez et al., 2010).

Another innovative aspect of the present study is the presentation of standardized variables with their relative value. This easily understandable information may be very useful to understand how the soccer players “manage” their time with regard to their overall result for the 30 m sprint. It also makes it possible to compare participants or the performance of the same participant, at different moments in the year and to find out the percentage gain or loss experienced. In the 10–20 m section significant differences were found between the two groups with a Vmax10–20 of 8.45 ± 0.23 m·s^−1^ and 8.29 ± 0.28 m·s^−1^, *p* < 0.05, which was higher in the competitive players (Figure 1). In this section RV10–20 was approximately 95%. The results for the 0–10 m section show that Vmax0–10 was very nearly significant, so these players can be said to have achieved higher velocity peaks although the time over the whole 30 m was similar. The rest of the sections does not reveal significant differences although the competitive players obtained slightly higher maximum velocities. This could be explained by the fact that in matches the elite players perform more short sprints (0–10 m) than longer sprints (> 10 m) (Di Salvo et al., 2010). The fact that in the present study differences were found at distances under 20 m reinforces this concept. This can be important in different critical play situations, for instance, arriving first to gain possession of the ball or defend an offensive action thus achieving a better position.

Four different playing positions were compared, and the effect of the interaction between the competitive level and playing position was also analyzed among the eight groups (Figure 2). Significant differences were found in average velocity for the 0–10 m section for competitive players, (*p* < 0.05 and η^2^ = 0.21, a moderately consistent effect) (Figure 2). This shows that the competitive players revealed specificity of the position in relation to the average velocity variable in the 0–10 m section with, in order, first the forwards, followed by the central midfielders, wide defenders-midfielders and central defenders. In contrast the non-competitive players did not reveal a clearly defined difference according to the position. Similar data were found in another study of Spanish soccer players in the 30 m flat sprint where forwards were faster than defenders and goalkeepers (Gil et al., 2007), and in the s-league of Singapore where forwards performed significantly better in the running Repeat Sprint Ability test than midfielders and defenders (Aziz et al., 2008). However, these differences were not found in the Turkish league (Taskin, 2008). It could be said, with a certain degree of consistency, that average velocity over the first 10 m defines specificity of the position in competitive soccer players.

In conclusion, among competitive soccer players, the forwards were the fastest followed by the central midfielders, the wide defenders/midfielders and then the central defenders. These differences were not found among the non-competitive players. Average velocity over the first 10 m could be an indicator to be taken into account when assigning playing positions for competitive soccer players. The 10 m test could be a good method for identifying forwards and central midfielders to determine which players will be most effective in each position. The competitive players reached higher maximum velocities in the 10–20 m section than did the non-competitive players. The use of an accurate system such as the laser system permitted recording of velocity variables and the position of the athletes in real time with a sampling frequency of 200 Hz and accuracy of ± 0.06 m, providing a more sensitive analysis of the velocity curves.

This study provides data that can be used by coaches to make a more complete assessment of their players’ physical level profiles with regard to the quality of their velocity. This may help coaches to determine the strengths and weaknesses of their players to place training emphasis on improving the weaker variables. Moreover, the presentation of standardized variables could facilitate understanding of the results and the comparison of inter-subject and intra-subject data at different times of the year to record the percentage gains and losses in velocity experienced over the competitive season.

## Figures and Tables

**Table 1:** Descriptive characteristics of the soccer players participating in the study

	n	Age (years)	Body mass (kg)	Body height (m)	Experience	
					Competition^*^ (years)	Non competition^**^ (years)
**Level of competition**						
Competitors	21	21.14 ± 1.62	73.70 ± 7.77	1.78 ± 0.05	2.76 ± 0.88	--
Non competitors	21	21.23 ± 1.84	71.65 ± 6.72	1.77 ± 0.06	--	3.07 ± 0.34
**Playing positions**						
Central defenders	11	21.25 ± 1.93	77.70 ± 6.93	1.81 ± 0.05	4.00 ± 2.12	2.60 ± 1.14
Wide def-midfield	13	21.46 ± 1.70	71.38 ± 6.20	1.76 ± 0.10	2.71 ± 1.25	3.67 ± 1.03
Central midfielders	11	21.00 ± 1.79	71.05 ± 6.41	1.75 ± 0.05	2.33 ± 0.82	2.75 ± 0.50
Forwards	7	21.00 ± 0.58	71.71 ± 7.25	1.79 ± 0.06	2.00 ± 0.00	3.25 ± 0.50

* Period of time, prior to this study, participating in competition.

** Period of time, prior to this study, without participating in competition

**Table 2:** Descriptive data of average velocities by the sections of the soccer players participating in the study

	Playing Position	Level of competition								

		NON COMPETITORS			COMPETITORS			TOTAL		

		Average	SD	N	Average	SD	N	Average	SD	N
Vm 0–10 (m·s^−1^)	Central defender	5.52	.03	6	5.38	.17	5	5.46	.13	11
	Wide def-midfield.	5.50	.12	6	5.47	.20	7	5.48	.16	13
	Central midfielder	5.45	.14	5	5.63	.16	6	5.55	.17	11
	Forward	5.41	.28	4	5.65	.18	3	5.51	.26	7
	Total	5.48	.15	21	5.52	.20	21	5.50	.17	42
Vm 10–20 (m·s^−1^)	Central defender	7.90	.17	6	7.71	.40	5	7.81	.30	11
	Wide def-midfield.	7.83	.25	6	7.95	.27	7	7.90	.26	13
	Central midfielder	7.82	.23	5	8.06	.19	6	7.95	.23	11
	Forward	7.68	.38	4	7.95	.14	3	7.80	.31	7
	Total	7.82	.25	21	7.93	.29	21	7.87	.27	42
Vm 20–30 (m·s^−1^)	Central defender	8.21	.23	6	8.14	.54	5	8.18	.38	11
	Wide def-midfield.	8.32	.35	6	8.37	.45	7	8.35	.39	13
	Central midfielder	8.14	.45	5	8.51	.18	6	8.35	.37	11
	Forward	8.14	.41	4	8.42	.27	3	8.26	.36	7
	Total	8.21	.34	21	8.36	.39	21	8.29	.37	42
Vm 0–20 (m·s^−1^)	Central defender	6.49	.06	6	6.32	.25	5	6.41	.18	11
	Wide def-midfield.	6.45	.16	6	6.47	.22	7	6.46	.19	13
	Central midfielder	6.41	.17	5	6.62	.16	6	6.53	.19	11
	Forward	6.34	.31	4	6.60	.14	3	6.45	.27	7
	Total	6.43	.17	21	6.50	.22	21	6.46	.20	42
Vm 0–30 (m·s^−1^)	Central defender	6.97	.09	6	6.82	.32	5	6.90	.22	11
	Wide def-midfield.	6.97	.20	6	6.99	.26	7	6.98	.23	13
	Central midfielder	6.89	.24	5	7.14	.17	6	7.03	.23	11
	Forward	6.83	.34	4	7.10	.16	3	6.95	.29	7
	Total	6.92	.21	21	7.01	.26	21	6.97	.23	42
Vm 10–30 (m·s^−1^)	Central defender	8.03	.16	6	7.90	.46	5	7.97	.32	11
	Wide def-midfield.	8.05	.29	6	8.14	.34	7	8.10	.31	13
	Central midfielder	7.96	.33	5	8.27	.18	6	8.13	.29	11
	Forward	7.89	.39	4	8.16	.20	3	8.01	.33	7
	Total	7.99	.28	21	8.12	.33	21	8.06	.31	42

**Table 3:** Descriptive data of maximum velocities by the sections, in absolute and relative values, of the soccer players participating in the study

	Playing Position	Level of competition								

		NON COMPETITORS			COMPETITORS			TOTAL		

		Average	SD	N	Average	SD	N	Average	SD	N
Vmax 0–10 (m·s^−1^)	Central defender	7.41	.23	6	7.35	.32	5	7.38	.26	11
	Wide def-midfield.	7.40	.26	6	7.61	.33	7	7.52	.31	13
	Central midfielder	7.28	.34	5	7.58	.22	6	7.44	.31	11
	Forward	7.23	.31	4	7.39	.10	3	7.30	.24	7
	Total	7.34	.27	21	7.51	.28	21	7.43	.29	42
RV 0–10 (%)	Central defender	84.97	4.38	6	83.11	5.47	5	84.13	4.75	11
	Wide def-midfield.	84.39	4.07	6	87.34	2.93	7	85.98	3.68	13
	Central midfielder	82.36	3.18	5	85.06	1.74	6	83.83	2.75	11
	Forward	83.13	3.03	4	79.98	4.78	3	81.78	3.88	7
	Total	83.83	3.67	21	84.63	4.25	21	84.23	3.94	42
Vmax 10–20 (m·s^−1^)	Central defender	8.35	.14	6	8.39	.25	5	8.37	.19	11
	Wide def-midfield.	8.33	.27	6	8.42	.27	7	8.38	.26	13
	Central midfielder	8.28	.33	5	8.54	.22	6	8.42	.30	11
	Forward	8.15	.44	4	8.48	.21	3	8.29	.38	7
	Total	8.29	.29	21	8.46	.24	21	8.37	.27	42
RV 10–20 (%)	Central defender	95.78	4.61	6	94.85	4.94	5	95.36	4.54	11
	Wide def-midfield.	94.96	3.21	6	96.65	2.76	7	95.87	2.98	13
	Central midfielder	93.61	2.26	5	95.82	1.09	6	94.82	1.99	11
	Forward	93.56	1.75	4	91.80	5.97	3	92.81	3.78	7
	Total	94.61	3.21	21	95.29	3.69	21	94.95	3.43	42
Vmax 20–30 (m·s^−1^)	Central defender	8.74	.33	6	8.87	.65	5	8.80	.48	11
	Wide def-midfield.	8.78	.39	6	8.72	.31	7	8.75	.33	13
	Central midfielder	8.85	.44	5	8.92	.29	6	8.88	.35	11
	Forward	8.71	.50	4	9.26	.70	3	8.95	.61	7
	Total	8.77	.38	21	8.89	.46	21	8.83	.42	42

## References

[b1-jhk-44-143] Adamczewski H, Perlt B (1997). Run-Up Velocities of Female and Male Pole Vaulting and some Technical Aspects of Women's Pole Vault. New Stud Athlet.

[b2-jhk-44-143] Aziz AR, Mukherjee S, Chia MYH, Teh KC (2008). Validity of the Running Repeated Sprint Ability Test among Playing Positions and Level of Competitiveness in Trained Soccer Players. Int J Sports Med.

[b3-jhk-44-143] Barbero-Álvarez JC, Coutts A, Granda J, Barbero-Álvarez V, Castagna C (2010). The Validity and Reliability of a Global Positioning Satellite System Device to Assess Speed and Repeated Sprint Ability (RSA) in Athletes. J Sci Med Sport.

[b4-jhk-44-143] Brüggemann GP, Koszewski D, Müller H (1999). Biomechanical Research Project. Athens 1997: Final report.

[b5-jhk-44-143] Cometti G, Maffiuletti NA, Pousson M, Chatard JC, Maffulli N (2001). Isokinetic Strength and Anaerobic Power of Elite, Subelite and Amateur French Soccer Players. Int J Sports Med.

[b6-jhk-44-143] Delecluse C, Roelants M, Diels R, Koninckx E, Verschueren S (2005). Effects of Whole Body Vibration Training on Muscle Strength and Sprint Performance in Sprint-Trained Athletes. Int J Sports Med.

[b7-jhk-44-143] Di Salvo V, Baron R, González-Haro C, Gormasz C, Pigozzi F, Bachl N (2010). Sprinting Analysis of Elite Soccer Players during European Champions League and UEFA Cup Matches. J Sport Sci.

[b8-jhk-44-143] Di Salvo V, Baron R, Tschan H, Calderon Montero FJ, Bachl N, Pigozzi F (2007). Performance Characteristics According to Playing Position in Elite Soccer. Int J Sports Med.

[b9-jhk-44-143] Di Salvo V, Collins A, McNeill B, Cardinale M (2006). Validation of Prozone^®^: A New Video-Based Performance Analysis System. Int J Perf Anal Spor.

[b10-jhk-44-143] Di Salvo V, Gregson W, Atkinson G, Tordoff P, Drust B (2009). Analysis of High Intensity Activity in Premier League Soccer. Int J Sports Med.

[b11-jhk-44-143] Dickwach H, Hildebrand F, Perlt B (1994). A Laser Velocity Measuring Device. The determination of velocity courses in the jumping events with the use of the LAVEG measuring device. New Stud Athlet.

[b12-jhk-44-143] Faude O, Koch T, Meyer T (2012). Straight sprinting is the most frequent action in goal situations in professional football. J Sport Sci.

[b13-jhk-44-143] Ferro A (2012). BioLaserSport. Trademark n° 3019808/9.

[b14-jhk-44-143] Ferro A, Floría P (2010). Kinematic analysis system in real time for the training and the sports competitions.

[b15-jhk-44-143] Ferro A, Floria P, Villacieros J, Aguado R (2012). Validity and reliability of the laser sensor of BioLaserSport^®^ system for the analysis of the running velocity. Rev Int Cien Deporte.

[b16-jhk-44-143] Gil SM, Gil J, Ruiz F, Irazusta A, Irazusta J (2007). Physiological and Anthropometric Characteristics of Young Soccer Players According to their Playing Position: Relevance for the Selection Process. J Strength Cond Res.

[b17-jhk-44-143] Harrison AJ, Jensen RL, Donoghue O (2005). A Comparison of Laser and Video Techniques for Determining Displacement and Velocity during Running. Meas Phys Educ Exerc Sci.

[b18-jhk-44-143] Kaplan T (2010). Examination of Repeated Sprinting Ability and Fatigue Index of Soccer Players According to their Positions. J Strength Cond Res.

[b19-jhk-44-143] Kaplan T, Erkmen N, Taskin H (2009). The Evaluation of the Running Speed and Agility Performance in Professional and Amateur Soccer Players. J Strength Cond Res.

[b20-jhk-44-143] Krustrup P, Mohr M, Ellingsgaard H, Bangsbo J (2005). Physical Demands during an Elite Female Soccer Game: Importance of Training Status. Med Sci Sport Exer.

[b21-jhk-44-143] Mohr M, Krustrup P, Bangsbo J (2003). Match Performance of High-Standard Soccer Players with Special Reference to Development of Fatigue. J Sport Sci.

[b22-jhk-44-143] Rampinini E, Bishop D, Marcora SM, Ferrari Bravo D, Sassi R, Impellizzeri FM (2007). Validity of Simple Field Tests as Indicators of Match-Related Physical Performance in Top-Level Professional Soccer Players. Int J Sports Med.

[b23-jhk-44-143] Rampinini E, Sassi A, Morelli A, Mazzoni S, Fanchini M, Coutts AJ (2009). Repeated-Sprint Ability in Professional and Amateur Soccer Players. Appl Physiol Nutr Me.

[b24-jhk-44-143] Reilly T, Thomas V (1976). Motion Analysis of Work-Rate in Different Positional Roles in Professional Football Match-Play. J Hum Movement Stud.

[b25-jhk-44-143] Reilly T, Williams AM, Nevill A, Franks A (2000). A Multidisciplinary Approach to Talent Identification in Soccer. J Sport Sci.

[b26-jhk-44-143] Sayers A, Sayers BE, Binkley H (2008). Preseason Fitness Testing in National Collegiate Athletic Association Soccer. Strength Cond J.

[b27-jhk-44-143] Taskin H (2008). Evaluating Sprinting Ability, Density of Acceleration, and Speed Dribbling Ability of Professional Soccer Players with Respect to their Positions. J Strength Cond Res.

[b28-jhk-44-143] Wisloff U, Castagna C, Helgerud J, Jones R, Hoff J (2004). Strong Correlation of Maximal Squat Strength with Sprint Performance and Vertical Jump Height in Elite Soccer Players. Brit J Sport Med.

[b29-jhk-44-143] Zwierko T, Lesiakowski P (2007). Selected Parameters of Speed Performance of Basketball Players with Different Sport Experience Levels. Stud Phys Cult Tourism.

